# Open‐Source and Commercial 3‐Dimensional Computed Tomography Methods Show Limited Agreement for Classifying Acetabular Morphology in Patients Undergoing Hip Arthroscopy

**DOI:** 10.1002/ars2.70148

**Published:** 2026-11-03

**Authors:** Ashley A. Ellingwood, Rajvarun S. Grewal, Henry M. Gass, Alisha Chen, Daniel Woo, Byron V. L. Beasley, James D. Bomar, Kevin C. Parvaresh, Vidyadhar V. Upasani, Matthew R. Schmitz

**Affiliations:** ^1^ Division of Orthopedics Rady Children's Hospital‐San Diego San Diego California U.S.A.; ^2^ California Health Sciences University Clovis California U.S.A.; ^3^ Hoag Orthopedic Institute Irvine California U.S.A.; ^4^ Department of Orthopaedic Surgery University of California San Diego San Diego California U.S.A.

## Abstract

**Purpose:**

To compare acetabular morphology classifications derived from a validated open‐source (freely available) method with those derived from commercially available software in patients undergoing hip arthroscopy for femoroacetabular impingement syndrome.

**Methods:**

We analyzed preoperative 3‐dimensional computed tomography imaging in patients with femoroacetabular impingement syndrome using open‐source and commercial software. The open‐source method calculated acetabular coverage across 5 regions and generated *z*‐scores relative to an internal normative database. Hips with *z*‐scores ≥2 or ≤−2 were classified as abnormal. The commercial method categorized hips as normal, undercovered, or overcovered based on published thresholds (lateral center‐edge angle, Tönnis angle, coverage percentage, and crossover sign) and focal depth mapping. The primary outcome was categorical agreement between methods.

**Results:**

Seventy‐six hips (55 women, 21 men; mean age 35.5 ± 12.3 years) were included. The open‐source method classified 58/76 hips as normal, 10/76 as dysplastic, and 8/76 as overcovered. The commercial method classified 14/76 hips as normal, 29/76 as undercovered, and 33/76 as overcovered. A total of 23/76 hips were classified the same in both systems and called agreed. Disagreements (53/76 hips) fell into 3 categories: (1) essentially similar despite different labels because of borderline *z*‐scores or small focal areas (n = 24), (2) disagreements driven by Tönnis angle alone (n = 8), and (3) true disagreements (n = 21). Notably, 5 hips (6.6%) showed opposite classifications (e.g., dysplastic vs overcovered). Subanalysis of *z*‐scores showed that 69.8% of disagreements reflected borderline morphology (*z*‐scores of ±1‐2).

**Conclusions:**

Although both open‐source and commercial methods provide 3‐dimensional classification of acetabular morphology, categorical agreement was limited. Most disagreements reflected borderline morphology or small focal areas of uncertain clinical relevance. A minority showed true classification opposites.

**Clinical Relevance:**

Awareness of the methodological differences between open‐source and commercial 3‐dimensional computed tomography platforms may help surgeons appropriately interpret acetabular morphology classifications during preoperative planning for patients undergoing hip arthroscopy for femoroacetabular impingement syndrome.

## Graphical abstract

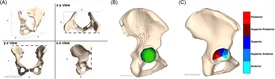

Three‐dimensional (3D) evaluation of acetabular morphology has become increasingly valuable for the diagnosis and surgical planning of hip dysplasia and femoroacetabular impingement syndrome (FAIS). Traditional radiographs are limited by projection variability and pelvic tilt, which can distort measurements such as the lateral center‐edge angle (LCEA) and Tönnis angle.^1^, ^2^, ^3^ Additionally, prior work has shown that the pelvic position in the sagittal plane significantly alters apparent acetabular coverage.^3^ Computed tomography (CT) can provide a more comprehensive assessment of hip morphology, including acetabular orientation, regional coverage, and femoral and acetabular version and torsion, compared with static radiographs.

With the growing use of 3D imaging in clinical practice, software has been developed that allows clinicians to quantify the acetabular morphology. Commercial methods now generate automated reports that include parameters such as LCEA, version, Tönnis angle, coverage percentage, and highlight focal areas deemed abnormal based on population‐derived confidence intervals (CIs).^4^ A validated open‐source (freely available) method has also been created to calculate coverage across regional quadrants and generate *z*‐scores directly relative to a normative CT database.^5^ This provides reproducible, patient‐specific quantification of acetabular coverage and addresses the limitations of 2‐dimensional radiographs and subjective 3D interpretation. Despite these advances, direct comparisons between commercial and open‐source methods remain limited, and variability often exists in classification thresholds across platforms, databases, and imaging modalities.^6^, ^7^ For example, commercial software uses categorical thresholds (e.g., LCEA ≥40° for overcoverage and LCEA ≤18° for undercoverage) derived primarily from radiographic literature rather than large 3D normative cohorts and may not consistently align with *z*‐score‐based measurements.^2^, ^4^, ^8^, ^9^, ^10^, ^11^ As a result, the same hip may be categorized differently depending on the chosen program.

The purpose of this study was to compare acetabular morphology classifications derived from a validated open‐source (freely available) method with those derived from commercially available software in patients undergoing hip arthroscopy for FAIS. We hypothesized that the 2 systems would show agreement in most cases but that categorical thresholds may lead to classification variability in borderline hips.

## METHODS

### Study Population and Data Collection

After obtaining Institutional Review Board approval (#20235759), a retrospective review of patients who were diagnosed with FAIS at a single orthopaedic hospital (Hoag Orthopedic Institute) and obtained preoperative pelvic CT imaging between September 2020 and May 2023 was performed. Individuals were eligible for inclusion if they had complete preoperative imaging, consisting of a standard anteroposterior pelvis radiograph, a false‐profile hip radiograph, and a CT scan obtained using the Stryker HipMap (Stryker, Mahwah, NJ) protocol.^12^, ^13^, ^14^, ^15^ Eighty‐four patients were excluded by Institution 1 (Hoag Orthopedic Institute) because their CT was obtained at an outside facility and was unavailable for processing. Two additional patients were excluded by Institution 2 (Rady Children's Hospital, San Diego, CA): one because only a CT of the hemipelvis was obtained and the open‐source methodology requires a full pelvic CT for orientation purposes, and the other was excluded because the femoral head was in contact with the acetabulum and the open‐source method was unable to differentiate the 2. Patients were considered candidates for hip arthroscopy if they had a diagnosis of FAIS, as defined by the Warwick Agreement, and had failed to improve after at least 12 weeks of physician‐directed nonoperative treatment, including activity modification, anti‐inflammatory medications, physical therapy, and/or injections.^16^ Pelvic CT imaging was ordered for all patients meeting surgical indications as the standard of care for definitive morphologic diagnosis, though some patients were unable to obtain imaging because of insurance, financial, or timing considerations.^17^

All existing CT scans obtained using the commercial software, Stryker HipMap, that otherwise met our inclusion criteria were securely transferred to a separate orthopaedic hospital (Rady Children's Hospital) and underwent analysis using the open‐source method.^4^, ^5^ The results of the open‐source analysis were then compared with the corresponding reports generated by the commercially available software that had been used during the patients’ clinical evaluation and treatment to see if they agreed on the morphology classification. All disagreements were identified by a single reviewer (A.A.E.) and verified by a second reviewer (M.R.S.). For each disagreement, the reviewers discussed the results to determine the reason for disagreement.

### Open‐Source Method

CT scans were segmented using Mimics Medical (v21.0, Materialise, Leuven, Belgium) to isolate the bony pelvic anatomy.^5^ 3D models were then exported as stereolithography files and processed with custom MATLAB (The MathWorks, Natick, MA) algorithms. Standard pelvic alignment included 3 anatomical planes: axial (*x*‐*y*), coronal (*y*‐*z*), and sagittal (*x*‐*z*) using the anterior superior iliac spine and pubic tubercle (Figure 1A). Each acetabulum was fitted with a best‐fit sphere, and a direction vector derived from surface normals was used to calculate the acetabular version and tilt (Figure 1B). Regional morphology was assessed by dividing the acetabulum into 5 clinically relevant octants (anterior, superior‐anterior, superior, superior‐posterior, and posterior) (Figure 1C). The coverage angles were calculated for each region relative to the sphere center and compared with an internal database of age‐ and sex‐matched normal hips. Regional *z*‐scores were generated, and values ≥2 or ≤−2 standard deviations from the normative values were considered abnormal. A reviewer applied these predefined classification criteria to the software‐generated outputs to assign each hip to a morphology category (normal, dysplastic, or overcovered), including regional subtypes (e.g., dysplastic‐posterior and overcovered‐posterior). Additional data collected included side, sex, age, total acetabular surface area (cm^2^), version, and LCEA for each patient. Patients were excluded if their femoral head was fused in the socket or if they had a unilateral CT. The open‐source method requires the full pelvis to orient the hips appropriately and cannot delineate the acetabulum from the femoral head when it is fused.

**FIGURE 1:**
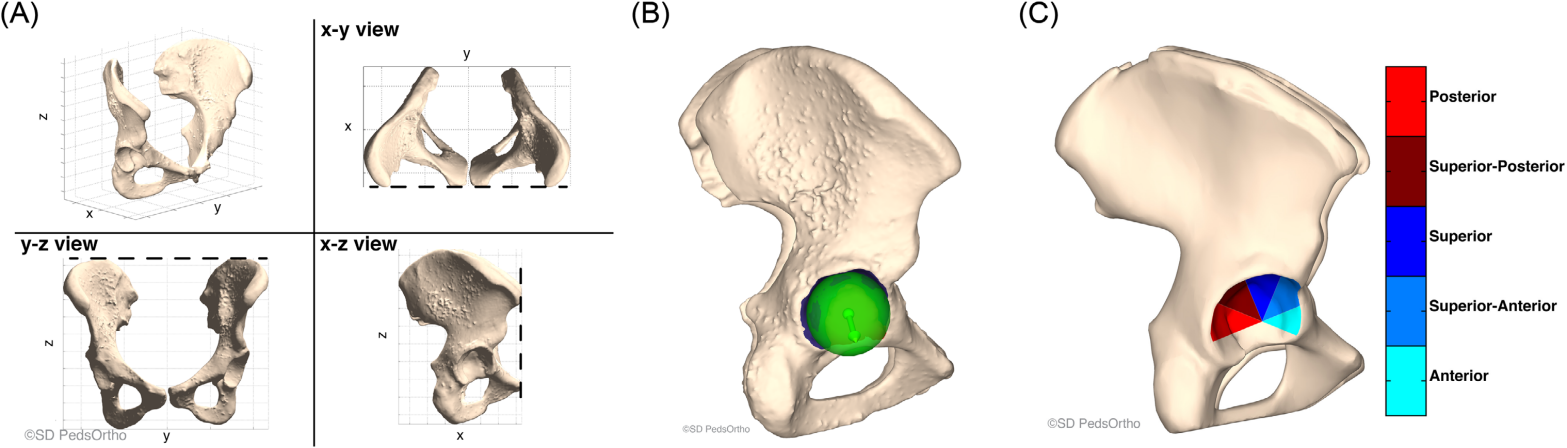
Pelvic alignment and regional coverage analysis: the 3D pelvis model is aligned in the axial (*x*‐*y*) plane by the ASIS, in the coronal (*y*‐*z*) plane by the iliac crests, and in the sagittal (*x*‐*z*) plane by the ASIS and pubic tubercle to ensure standardized alignment across patients (A). Each acetabulum is then modeled with a best‐fit sphere (green dot = center), from which a direction vector (arrow) is calculated based on averaged surface normals to determine acetabular version and tilt (B). For regional assessment, the acetabulum is subdivided into five 45° octants—anterior, superior‐anterior, superior, superior‐posterior, and posterior—and mean coverage angles are calculated relative to the sphere center, enabling the identification of areas of under‐ or overcoverage (C). (3D, 3‐dimensional; ASIS, anterior superior iliac spines.)

### Commercial Method

The commercially available software automatically aligns the pelvis to the coronal plane and generates a standardized report of acetabular and femoral morphology.^4^ Quantitative measurements include the LCEA, version, Tönnis angle, coverage percentage, femoral neck‐shaft angle, femoral torsion, alpha angles, and focal regions relative to a normative database. Commercial classifications followed the published criteria incorporated into the report's outputs. Hips were classified as overcovered if any of the following were present: LCEA >40°, Tönnis angle <0°, coverage >65%, crossover sign, or a lateral focal area. Hips were classified as undercovered (dysplastic) if LCEA <18° with 18° to 25° considered borderline, Tönnis angle >10°, coverage <50%, or a medial focal area. Hips were considered normal when none of the above criteria were met. A reviewer then applied these predefined classification criteria to the software‐generated outputs and focal coverage findings to assign each hip to a morphology category. When multiple morphologic findings were present, classifications were interpreted in the context of the overall acetabular morphology. Hips with an overall pattern of undercoverage and small focal areas of overcoverage were classified as undercovered, whereas hips without other abnormalities that showed focal findings meeting commercial criteria for abnormal morphology were classified according to those focal findings.

### Statistical Analysis

Basic descriptive statistics were reported. The unit of analysis was the hip. Direct comparisons were made between the 2 evaluation modalities for version and LCEA. Agreement among the 2 modalities for these 2 measurements was evaluated with the interclass correlation coefficient (ICC). ICCs were interpreted as outlined by Koo and Li, with <0.5 = poor, 0.5 to 0.75 = moderate, 0.75 to 09 = good, and >0.9 = excellent agreement.^18^ Each evaluation modality reports whether or not the hip was normal, overcovered, or undercovered. In the case of over‐ and undercoverage, the user is able to ascertain the location (anterior, superior, or posterior) of the over‐ or undercoverage. Basic descriptive statistics were used to report the rate at which the 2 modalities agreed with one another in regard to normality and over‐ or undercoverage, as well as the location of over‐ or undercoverage. Distribution of agreement rates among sex was evaluated with Fisher's exact test. No power analysis was performed because of the design of this study, which was to present the rate of qualitative agreement among the 2 evaluation modalities. The significance was defined as *P* < .05. Statistical analysis was performed using IBM SPSS Statistics (version 29).

## RESULTS

### Demographics

A total of 76 patients, with 55 women (72%) and 21 men (28%), were included in this study (Figure 2). There were 7 adolescent hips, which included patients 19 years old or younger. The mean age of the cohort was 35.5 years ± 12.3.

**FIGURE 2:**
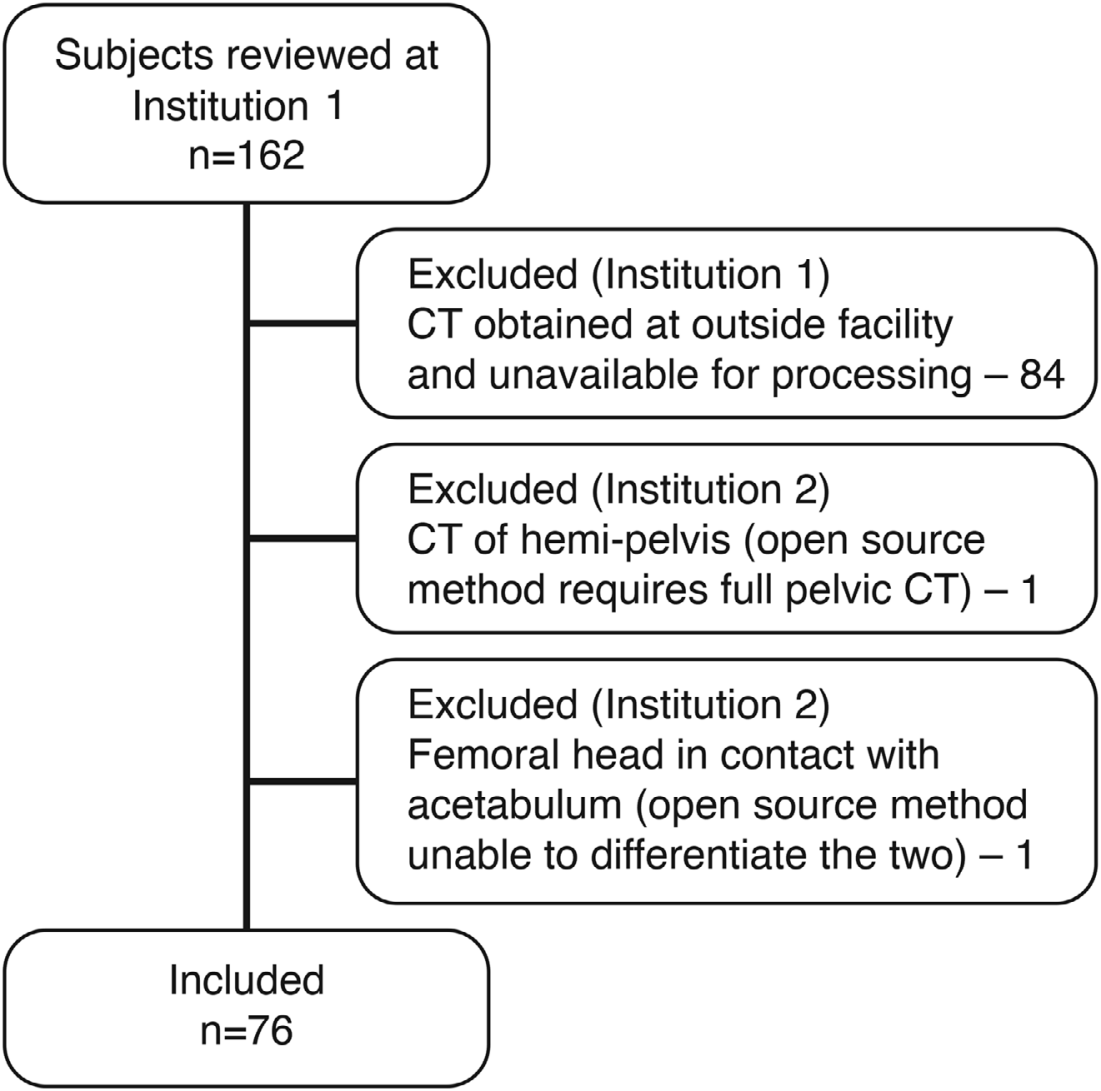
Flow diagram of exclusion criteria. (CT, computed tomography.)

### Open‐Source Method

Using the open‐source method, 58 hips (76.3%; 39 women, 19 men) were classified as normal, 10 (13.2%; 8 women, 2 men) as dysplastic, and 8 (10.5%; 8 women) as overcovered across regional subtypes (Table 1). Mean acetabular version was 23.4° ± 7.4, mean LCEA 32.0° ±7.1, mean tilt 41.3° ± 4.0, and average acetabular surface area 36.2 ± 9.8 cm^2^.

**TABLE 1:** Distribution of Classifications by Open‐Source and Commercial Methods

Classification	Open‐Source, N (%)	Commercial, N (%)
Normal	58 (76.3)	14 (18.4)
Dysplastic‐posterior	2 (2.6)	‐
Dysplastic‐superior	2 (2.6)	‐
Dysplastic‐superior/anterior	1 (1.3)	‐
Dysplastic‐anterior	5 (6.6)	‐
Overcovered‐anterior	1 (1.3)	‐
Overcovered‐posterior	3 (3.9)	‐
Overcovered‐superior	2 (2.6)	‐
Overcovered‐superior/posterior	2 (2.6)	‐
Undercovered (no subtype)	‐	29 (38.2)
Overcovered (no subtype)	‐	33 (43.4)
Total	76 (100)	76 (100)

### Commercial Method

The commercial method classified 14 hips (18.4%; 9 women, 5 men) as normal, 33 hips (43.4%; 26 women, 7 men) as overcovered, and 29 hips (38.2%; 20 women, 9 men) as undercovered (Table 1). The mean version was 22.0° ± 7.5°, and the mean LCEA was 31.9° ± 7.0°. Additional parameters included the alpha angle at 12 o'clock (49.3° ± 16.1°), femoral torsion (15.6° ± 13.5°, n = 75), and femoral neck‐shaft angle (129.5° ± 5.2°).

Two overlapping continuous variables measured by both methodologies were the acetabular version and LCEA. For the acetabular version, the ICC was 0.724 (95% CI: 0.597‐0.816, *P* < .001), indicating moderate reliability. For LCEA, the ICC was 0.867 (95% CI: 0.798‐0.914, *P* < .001), indicating good reliability.

### Agreement Between Methods

The 2 methods agreed on 23/76 hips (30.3%) (Table 2) with a marginally higher agreement rate among men (38%) than among women (27%); however, this difference in agreement rate was not statistically significant (*P* = .408). Of note, study identifier (ID) 103 was classified as dysplastic‐posterior by the open‐source method and undercovered by the commercial method; however, the commercial method also identified an anterior focal area of overcoverage (0‐3 mm in depth) that was not detected by the open‐source method.

**TABLE 2:** Hips With the Same Classifications Between Methods

Classification	Number of Hips (%)	Study IDs
Normal	12 (52.2)	1 (M), 5 (F), 11 (F), 46 (F), 47 (F), 64 (F), 66 (F), 71 (M), 72 (F), 76 (F), 83 (F), 84 (F)
Dysplastic	4 (17.4)	93 (M), 99 (F), 103 (M), 112 (M)
Overcovered	7 (30.4)	120 (M), 125 (F), 127 (M), 130 (F), 132 (M), 147 (F), 151 (F)
Total	23 (100)	‐

F, female; IDs, identifiers; M, male.

### Disagreements Between Methods

Disagreements occurred in 53 hips (69.7%) and were grouped into 3 categories.

#### Type 1: Essentially Similar (n = 24)

These hips were classified differently but pointed to the same clinical picture (Table 3). Six hips were called normal by open‐source and overcovered by commercial due solely to tiny focal areas ≤1 mm, whereas 2 hips (study IDs 29 and 74) showed similar findings with central focal areas extending beyond 0 to 1 mm depth. In 15 other hips that were classified as normal by open‐source, borderline *z*‐scores (±1‐2 SD) aligned with the commercial designation of undercovered. The last to note was 1 hip (Study ID 55) where the open‐source method classified the hip as overcoverage‐superior/posterior, but the commercial method labeled it normal; however, on closer review, the commercial findings appear borderline overcoverage with LCEA 40°, coverage 62%, and minute focal areas of borderline overcoverage.

**TABLE 3:** Essentially Similar Findings

Study IDs (Sex)	Open‐Source Classification	Commercial Classification
29 (M), 74 (F), 87 (F), 91 (M), 94 (F), 107 (F), 117 (F), 126 (F)	Normal	Overcovered
44 (M), 49 (F), 61 (F), 63 (M), 69 (F), 73 (M), 77 (F), 78 (F), 82 (F), 86 (F), 111 (F), 116 (M), 133 (F), 140 (F), 152 (M)	Normal	Undercovered
55 (M)	Overcovered	Normal

F, female; IDs, identifiers; M, male.

#### Type 2: Tönnis‐Driven (n = 8)

In all cases, elevated Tönnis angles on the commercial method produced undercovered classifications, whereas the open‐source method, which does not use CT‐derived Tönnis, classified the hips as normal.

#### Type 3: True Disagreements (n = 21)

These cases showed fundamentally different morphology, including 5 hips with opposite classifications (Table 4). All 5 hips with opposite classifications were labeled dysplastic by the open‐source method but overcovered by the commercial method. Study ID 51 was classified as dysplastic‐posterior by open‐source (posterior −2.3) but as overcovered by commercial because of a superior‐anterior focal area (0‐>3 mm). Similarly, study IDs 92 and 102 were both dysplastic‐anterior by open‐source (anterior ≤−2 SD) but overcovered by commercial only because of very small anterior focal areas (≤1 mm). In contrast, for study ID 123, open‐source detected dysplasia anteriorly with borderline superior‐posterior overcoverage (superior‐posterior 1.9, anterior −2.3), whereas the commercial method labeled it as overcovered based on LCEA 43° and a superior‐posterior focal area (0‐3 mm). Lastly, study ID 124 was classified as dysplastic‐anterior by the open‐source method (superior‐posterior 1.4, superior 1.3, anterior −2.4), but the commercial method classified it as overcovered based on LCEA 46°, Tönnis −1, and a superior‐anterior focal area (0‐3 mm).

**TABLE 4:** Cases of True Disagreements Between Methods

Study IDs (Sex)	Open‐Source Classification	Commercial Classification	Key Finding
51 (F)	Dysplastic‐posterior	Overcovered	Posterior deficiency vs superior‐anterior overcoverage
92 (M), 102 (F)	Dysplastic‐anterior	Overcovered	Severe anterior undercoverage missed by commercial; small anterior focal region
123 (F), 124 (F)	Dysplastic‐anterior	Overcovered	Anterior deficiency on open‐source vs overcoverage by commercial thresholds
7 (F)	Overcovered‐posterior	Normal	Posterior and superior‐posterior overcoverage detected only by open‐source
68 (F), 85 (F), 104 (F), 105 (M), 138 (F), 155 (F)	Normal	Overcovered	‐
20 (F), 28 (F), 40 (F), 48 (F), 54 (F), 60 (F), 141 (F), 158 (F), 160 (M)	Normal	Undercovered	‐

F, female; IDs, identifiers; M, male.

The remaining true disagreements involved the hips considered normal by the open‐source method but abnormal by the commercial method (Table 4). Six hips were classified as overcovered and 9 as undercovered by the commercial method, whereas 1 hip was classified as overcovered‐posterior by open‐source but normal by commercial. In 5 of these hips, the difference was driven primarily by focal areas identified by the commercial method that were not captured by regional *z*‐scores. Seven other hips showed mixed patterns, where open‐source reported borderline undercoverage, whereas the commercial system identified additional focal areas of overcoverage. Also, for 3 hips, differences were driven in part by Tönnis angle ≥11°, despite open‐source reporting borderline or even mild posterior overcoverage.

### Subanalysis of *Z*‐Scores

Among the hips classified overcovered by commercial but not open‐source (n = 27), 5 (18.5%) had maximum *z*‐scores between −1 and 1 SD, which is normal. Ten (37%) had maximum *z*‐scores between 1 and 2 SD, meaning borderline overcoverage. Seven (25.9%) had maximum *z*‐scores between −1 and −2 SD, meaning borderline undercoverage. The remaining 5 (18.5%) had *z*‐scores less than −2 SD, representing definite undercoverage.

For hips classified undercovered by commercial but not open‐source method (n = 24), 4 (16.7%) had maximum *z*‐scores between −1 and 1 SD, meaning normal. Seventeen (70.8%) had maximum *z*‐scores between −1 and −2 SD, suggesting borderline undercoverage. Three (12.5%) had maximum *z*‐scores between 1 and 2 SD, suggesting borderline overcoverage. The 2 hips classified as normal by commercial but abnormal by open‐source had maximum *z*‐scores >2 SD, consistent with definite overcoverage.

## DISCUSSION

The most important finding of this study was that the categorical agreement between the open‐source and commercial methods was limited, with most disagreements reflecting borderline morphology or small focal abnormalities rather than opposite classifications. The open‐source method classified 58/76 hips (76.3%) as normal, 10/76 (13.2%) as dysplastic or undercovered, and 8/76 (10.5%) as overcovered, whereas the commercial method classified 14/76 hips (18.4%) as normal, 29/76 (38.2%) as dysplastic or undercovered, and 33/76 (43.4%) as overcovered. When hip classifications were compared between the 2 systems, categorical agreement was limited. Overall, the 2 methods agreed in classification for only 23/76 hips (30.3%), whereas 53/76 hips (69.7%) were classified differently between the 2 methods. Twenty‐one out of seventy‐six hips (27.6%) showed true conflicting classifications, including 5 hips with opposite classifications. The open‐source method tended to identify global patterns of dysplasia or overcoverage, whereas the commercial method more frequently detected small focal abnormalities, often ≤1 mm in depth. These differences reflect each method's design, with one emphasizing global regional coverage and the other focusing on focal mapping. Our findings suggest that both offer complementary insights into the acetabular morphology.

One important difference between the 2 methods was how acetabular inclination was interpreted. The commercial method uses a CT‐derived Tönnis angle, whereas the open‐source method uses an acetabular tilt. Although both describe acetabular inclination, they are not interchangeable.^3^, ^19^ Prior studies have shown that CT‐based measurements tend to overestimate the Tönnis angle compared with radiographs, as much as a 4° difference, which suggests that direct translation of radiographic criteria to CT data may introduce bias.^20^ Likewise, other studies have shown that small variations in image orientation substantially affect the apparent Tönnis angle, reinforcing its dependence on orientation rather than true anatomy.^3^, ^21^ These disagreements highlight the need for caution when applying radiographic thresholds to CT‐derived values.

Our findings further align with prior literature, showing that classification outcomes depend heavily on the measurement system, normative data, and imaging modality. Larson et al. defined acetabular rim contours using CT‐based 95% CIs, forming the basis for the *z*‐score methodology used in the open‐source method,^7^ whereas more recent studies rely on fixed cutoffs, such as LCEA ≥ 39° to 40°, to define abnormality even though these thresholds were not derived from large 3D normative series.^9^, ^10^, ^11^, ^22^ This may explain why commercial classifications differed from open‐source findings at the 40° threshold. Chadayammuri et al. similarly reported that CT‐based LCEA measurements averaged about 2° greater than radiographs, especially among hips with combined dysplasia and FAIS, showing that imaging modality alone can shift the same hip across thresholds.^8^ Hanson et al. highlighted variability in anterior and LCEA depending on whether the sourcil or rim was used as the reference, and Falgout et al. showed that 2D radiographic measurements of LCEA and Tönnis angle differ significantly from corresponding 3D CT values. Together, these observations emphasize that thresholds must be used within the appropriate context.^2^, ^23^

Another source of disagreement was the commercial method's detection of very small focal areas (often ≤1 mm in depth), which may not always correspond to the overall acetabular morphology. In this study, focal findings were interpreted within the context of the overall acetabular morphology rather than considered in isolation. Hips with an overall pattern of undercoverage and small focal areas of overcoverage were classified as undercovered, whereas isolated focal abnormalities in otherwise normal hips contributed to abnormal commercial classifications. This distinction highlights the challenge of translating complex 3D acetabular morphology into simplified categorical classifications. Furthermore, the focal areas of coverage remain difficult to standardize. This study included patients up to 65 years old, and as a result, early degenerative changes or small osteophytes may mimic focal overcoverage on CT. Because CT cannot reliably distinguish subtle osteophytes from true morphology, some classifications may have been influenced by age. Smith et al. reported that 3D CT reconstructions detected more areas of overcoverage than radiographs, but many of the findings are subtle irregularities with unknown relevance.^17^ Functional outcomes may appear more strongly influenced by the overall extent of impingement rather than isolated lesions. For instance, Zhuo et al. associated residual overcoverage >20% with worse postoperative function, and Coughlin et al. reported smaller improvements in patients with global versus focal pincer deformities.^24^, ^25^ However, Matsuda et al. noted no difference in postoperative outcomes between global and focal pincer morphologies.^26^ Further research correlating imaging and patient outcomes is needed to determine whether such minor focal areas represent true sources of impingement or not.

Furthermore, many of the disagreements involved hips with borderline *z*‐scores (±1‐2 SD). Although these produced categorical differences, both systems pointed to the same underlying morphology. For example, borderline posterior overcoverage on open‐source often corresponds to small focal areas of overcoverage on commercial. Similarly, Graesser et al. used 3D CT to characterize acetabular morphology in patients with radiographically defined borderline dysplasia (LCEA 20°‐25°) and found significant variability in 3D coverage. They concluded that patients within this “borderline” range showed highly heterogeneous 3D deformities.^27^ These findings emphasize the importance of interpreting borderline classifications in the context of the broader clinical picture rather than relying solely on categorical thresholds.

True disagreements, where the 2 methods gave opposite classifications, were uncommon. In these cases, the open‐source method identified dysplasia, whereas the commercial method categorized the same hips as overcovered. These differences reflect the contrast between global and focal assessment. Larson et al. showed that regional and global measures of acetabular coverage can yield conflicting interpretations, showing that single‐parameter assessments may oversimplify complex morphology.^7^ Graesser et al. reported substantial heterogeneity in 3D coverage among hips classified as borderline dysplasia on radiographs, suggesting that such discordance may arise from analytic focus rather than true diagnostic inconsistency.^27^

In clinical practice, the choice of a method may depend on clinical priorities. For cases where global acetabular morphology is the key concern, such as evaluating dysplasia, retroversion, or generalized overcoverage, the open‐source method may be more informative. For cases where focal impingement is being considered, such as when planning for an arthroscopy, the commercial system's ability to dial in on small focal regions may be advantageous. Ideally, the use of both systems may provide a more comprehensive view. However, software‐based classifications should be viewed as adjunctive tools rather than independent determinants of surgical decision‐making. These findings should be interpreted in conjunction with the patient's clinical presentation, physical examination, standard radiographic evaluation, and surgeon judgment. A comprehensive assessment integrating multiple sources of information remains essential when determining the clinical significance of the acetabular morphology and planning treatment. Future studies should prospectively validate these methods against surgical outcomes and patient‐reported measures to determine the clinical relevance of borderline and small focal abnormalities and assess reproducibility across institutions.

### Limitations

This study has several limitations. First, it is a retrospective, single‐institution study, which may limit generalizability. Additionally, direct comparison between the 2 methods is imperfect and does not include the reproducibility of either modality, although the open‐source modality has published ICC for reproducibility at ICC >0.87 in subjects aged 8 to 19 years.^5^ Finally, differences in pelvic alignment, coordinate system definitions, and classification granularity between the open‐source and commercial software may limit direct comparability of the 2 approaches.

## CONCLUSIONS

Although both open‐source and commercial methods provide 3D classification of the acetabular morphology, categorical agreement was limited. Most disagreements reflected borderline morphology or small focal areas of uncertain clinical relevance. A minority showed true classification opposites.

## DISCLOSURES

The authors (A.A.E., R.S.G., H.M.G., A.C., D.W., B.V.L.B., J.D.B., K.C.P., V.V.U., M.R.S.) declare that they have no known competing financial interests or personal relationships that could have appeared to influence the work reported in this paper.
